# Magnetic resonance imaging findings of the posterior fossa in 47 patients with mucopolysaccharidoses: A cross‐sectional analysis

**DOI:** 10.1002/jmd2.12212

**Published:** 2021-03-21

**Authors:** Roberta Reichert, Juliano A. Pérez, Amauri Dalla‐Corte, Filippo Pinto e Vairo, Carolina F. M. de Souza, Roberto Giugliani, Gustavo R. Isolan, Marco Antonio Stefani

**Affiliations:** ^1^ Graduate Program in Medicine: Surgical Sciences Universidade Federal do Rio Grande do Sul (UFRGS) Porto Alegre Brazil; ^2^ Hospital de Clínicas de Porto Alegre (HCPA) Porto Alegre Brazil; ^3^ Universidade do Vale do Rio dos Sinos São Leopoldo Brazil; ^4^ Center for Individualized Medicine and Department of Clinical Genomics Mayo Clinic Rochester Minnesota USA; ^5^ Graduate Program in Medicine: Surgical Sciences UFRGS Porto Alegre Brazil

**Keywords:** mucopolysaccharidoses, posterior fossa, magnetic resonance imaging

## Abstract

**Background:**

Mucopolysaccharidoses (MPS) is a group of hereditary multisystemic lysosomal disorders. Most neuroimaging studies in MPS have focused on the supratentorial compartment and craniocervical junction abnormalities, and data regarding posterior fossa findings are scarce in the literature. Thus, our purpose is to describe posterior fossa findings on magnetic resonance imaging (MRI) of MPS patients.

**Methods:**

We reviewed routine MRI scans of MPS patients being followed up at our institution (types I, II, III, IV, and VI), focusing on posterior fossa structures.

**Results:**

Forty‐seven MPS patients were included. MRI‐visible perivascular spaces were commonly found in the midbrain and adjacent to the dentate nuclei (85% and 55% of patients, respectively). White‐matter lesion was not identified in most cases. Its most frequent localizations were in the pons and cerebellum (34% and 30% of patients, respectively). Enlargement of cerebrospinal fluid (CSF) spaces in the posterior fossa was present in 55% of individuals and was more frequent in neuronopathic patients (73% vs 40%; *P* = .02). Cerebellar volume was classified as normal, apparent macrocerebellum, atrophic, and hypoplastic in 38%, 38%, 21%, and 3% of patients, respectively. A depression of the posterior fossa floor in the midline sagittal plane was found in 22 patients (47%), which was statistical significantly associated with enlargement of CSF spaces (*P* = .02) and with apparent macrocerebellum (*P* = .03).

**Conclusion:**

The present study compiled the main posterior fossa findings in MPS patients. Classically described in the supratentorial compartment, MRI‐visible perivascular spaces, white matter lesions, and enlarged perivascular spaces were also found in the posterior fossa. However, atrophy, which commonly affects cerebral hemispheres, was not the most frequent cerebellar morphology found in our study. Moreover, potential findings for future research were described.

## 1 BACKGROUND

Mucopolysaccharidoses (MPS) is a group of hereditary lysosomal disorders with an estimated incidence of 1 per 25 000 individuals.^1^ In MPS, genetic variants lead to deficiency in lysosomal enzymes, which are involved in the degradation of glycosaminoglycans leading to accumulation of these partially degraded macromolecules within lysosomes and in the extracellular space, culminating in cell dysfunction of several organs and body tissues. According to the deficient lysosomal enzyme, MPS are classified into seven types (I, II, III, IV, VI, VII, and IX). Most of them are inherited in an autosomal recessive pattern, except for type II, which is an X‐linked disease.^2^ Clinically, MPS are multisystem, heterogeneous disorders, with vary clinical presentations and severity.^3^

Individuals with MPS I, II, III, VII, and IX might present with neurological impairment. However, even the individuals with the non‐neuropathic forms might have brain abnormalities. Magnetic resonance imaging (MRI) is the modality of choice for neuraxial evaluation in MPS, as in other hereditary metabolic diseases.^4^ White matter lesions, cortical atrophy, hydrocephalus, enlarged perivascular spaces, and stenosis of the craniocervical junction are the main imaging findings described in this population. These abnormalities occur in different combinations and severities, even in individuals with the same MPS type.^5^, ^6^, ^7^ Most neuroimaging studies in MPS have focused on the supratentorial compartment and craniocervical junction.^4^, ^8^, ^9^, ^10^, ^11^ Studies specifically describing posterior fossa findings in these patients are scarce. White matter lesions and enlarged perivascular spaces have been described in the brainstem and cerebellum. Abnormal cerebellar volume (hypoplasia and macrocerebellum), mega cisterna magna, arachnoid cyst, and Chiari I malformation are other reported findings.^12^, ^13^, ^14^, ^15^

Within this context, this study aims to describe the findings observed in routine brain MRI studies of patients with MPS, focusing on posterior fossa structures.

## 2 MATERIAL AND METHODS

### 2.1 Study design and participants

The study was approved by the Ethics Committee of our Hospital de Clínicas de Porto Alegre, Brazil. The cohort is composed of patients with MPS who are followed in the Medical Genetics Service at our institution. Informed consent was obtained from all subjects or legal guardian. All methods were carried out in accordance with relevant guidelines and regulations.

This cross‐sectional study included all patients with confirmed MPS followed at our institution who had undergone routine brain MRI, usually performed at the beginning of outpatient follow‐up, from October 2012 to the time of writing. The inclusion criteria were: (a) confirmed diagnosis of MPS by enzyme assay and/or DNA testing, including all MPS types, with no age restriction (adult and pediatric patients); and (b) available brain MRI on picture archiving system, including at least axial fluid‐attenuated inversion recovery (FLAIR) and T2‐weighted images and three‐dimensional T1‐weighted images. Patients for whom brain MRI was not available on picture archiving system were excluded. All MRI studies were performed in a 1.5T scanner (Achieva, Philips, Best, Netherlands).

### 2.2 Image analysis and variables of interest

Two experienced neuroradiologists evaluated the images by consensus. The following variables were analyzed in the posterior fossa:

#### 2.2.1 MRI‐visible perivascular spaces

Defined as cystic structures isointense to cerebrospinal fluid (CSF) in all pulse sequences. MRI‐visible perivascular spaces were classified as absent or present. If present, the maximum axial diameter was measured and the location was described (midbrain, pons, medulla, and/or cerebellum).

#### 2.2.2 White matter lesions

Characterized by focal or confluent T2‐FLAIR hyperintense, T1‐hypointense lesions in white matter areas. Presence and location were described.

#### 2.2.3 Cerebellar volume

The cerebellar volume was evaluated qualitatively on sagittal and axial images and classified as normal, atrophic (enlarged cerebellar sulci), hypoplastic (reduced cerebellar dimensions, but preserved morphology), or apparent macrocerebellum (increased cerebellar dimensions with reduction or absence of pericerebellar CSF), following a previously described model for MPS patients.^12^

#### 2.2.4 Middle cerebellar peduncles width

**FIGURE 1:**
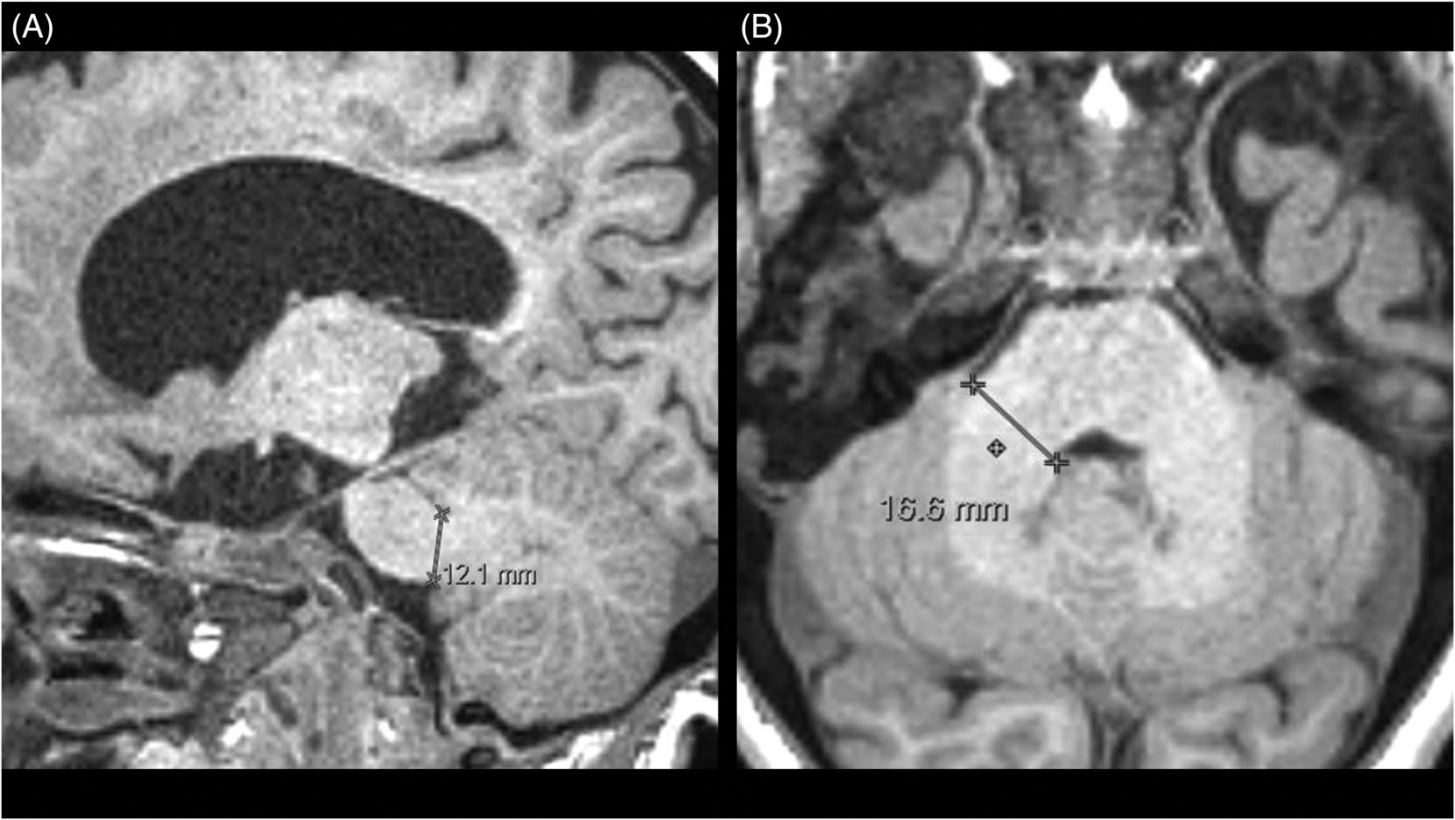
A, Sagittal T1‐weighted volumetric acquisition demonstrating measurement of middle cerebellar peduncle width (distance between its superior and inferior borders). B, Axial T1‐weighted volumetric acquisition demonstrating measurement of middle cerebellar peduncle width, perpendicular to its anteroposterior axis

The evaluators subjectively perceived a thickened aspect of the middle cerebellar peduncles (MCPs) in several patients. This finding has not been described previously in patients with MPS, and, to our knowledge, there is no standardized measurement for these structures. Then we decided to use as reference the measurement method described in an article that evaluated MCP width in patients with multiple system atrophy, Parkinson disease, and control subjects.^16^ Right and left MCPs were identified on parasagittal view and the distance between their superior and inferior borders was measured (Figure 1A). The largest laterolateral diameter of MCPs on axial slices, perpendicular to their anteroposterior axis, was also measured (Figure 1B). Right and left MCPs were measured separately, and a mean value for both MCPs was calculated.

#### 2.2.5 Posterior fossa shape

**FIGURE 2:**
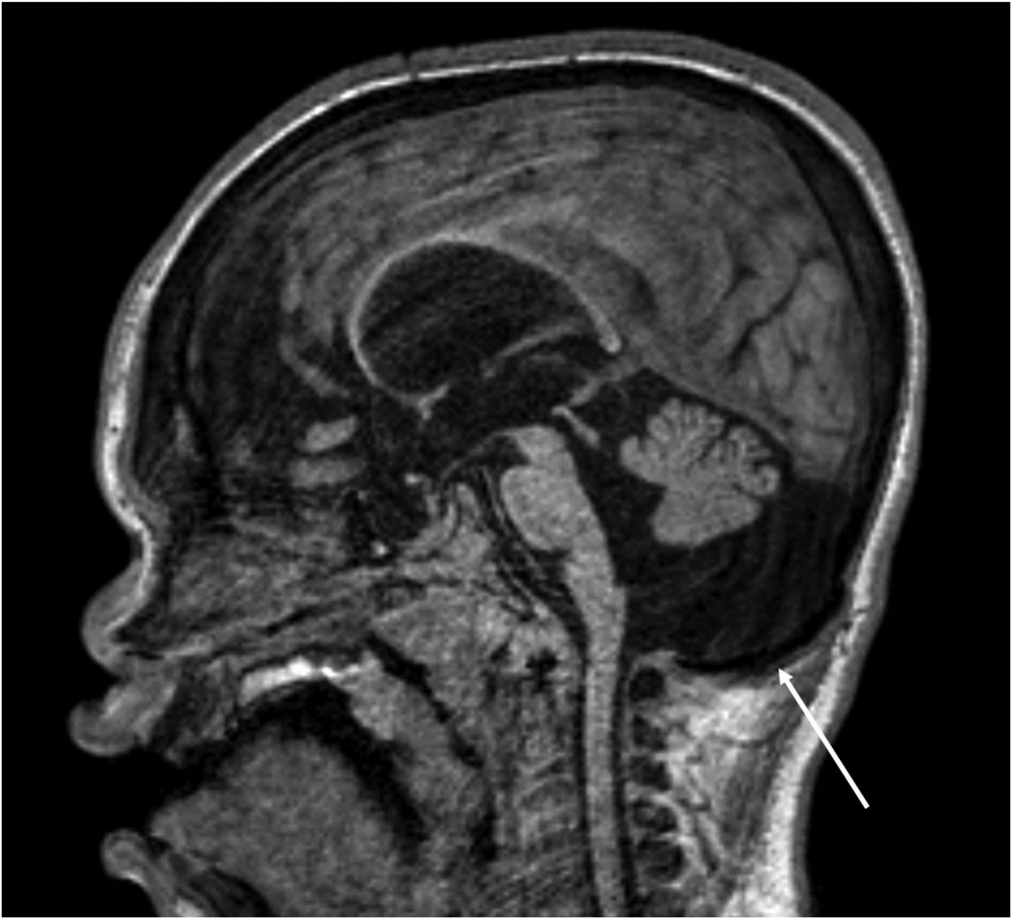
MPS IIA in a 7‐year‐old boy. Sagittal T1‐weighted volumetric acquisition showing depression of the posterior fossa floor in the midline sagittal plane (arrow), a previously undescribed abnormality we termed “inverted J‐shaped posterior fossa.” MPS, mucopolysaccharidoses

Some patients in our series were found to have a hook‐shaped depression of the posterior fossa floor in the midline sagittal plane (Figure 2). To the best of our knowledge, there is no previous description of this finding in the literature. We denominated this morphologic alteration “inverted J‐shaped posterior fossa,” and it was classified as absent or present.

#### 2.2.6 Enlargement of CSF spaces

Originally, we intended to assess the presence of mega cisterna magna and arachnoid cyst individually. However, the evaluators realized it was not possible to clearly distinguish between these two entities in several patients, due to their small size and similar location. Thus, mega cisterna magna and arachnoid cyst were clustered into one category: enlargement of CSF spaces. This was classified as absent or present, and, if present, bilateral or unilateral.

#### 2.2.7 Chiari malformation

Caudal descent of the cerebellar tonsils and brain stem through the foramen magnum was also recorded as absent or present.

In addition to the evaluation of brain MRI, clinical data (MPS type, gender, and age at MRI scan) were collected from all included patients.

### 2.3 Statistical methods

Variables were described for the whole population of patients and for each MPS type group separately. Due to the wide age variation among patients, this parameter was presented as a range between the youngest and the oldest individuals. Other continuous variables were described as mean and SD. All categorical variables were presented as absolute and relative (percentage) frequencies. Chi‐square test and *t* test were used to compare categorical and continuous variables, respectively.

## 3 RESULTS

### 3.1 Patients

Forty‐seven MPS patients (29 males and 18 females) met the inclusion criteria and were enrolled in the present study. In this population, there were 13 patients with MPS type I, 15 with MPS type II, 6 with MPS type III, 10 with MPS type IV, and 3 with MPS type VI. There were no cases of MPS type VII or IX. The age of the patients at the time of brain MRI scan ranged from 0.8 to 36.9 years. These descriptive data and MPS subtypes are summarized in Table 1.

**TABLE 1:** Clinical and demographic data

Parameter	MPS type I (n = 13)	MPS type II (n = 15)	MPS type III (n = 6)	MPS type IV (n = 10)	MPS type VI (n = 3)	Total (n = 47)
MPS subtype (n)	Hurler: 8 Hurler‐Scheie: 2 Scheie: 3	IIA: 8 IIB: 7	IIIA: 1 IIIB: 4 IIIC: 1 IIID: 0	IVA: 10 IVB: 0	Not applicable	Not applicable
Male sex (n)	7 (54%)	15 (100%)	3 (50%)	2 (20%)	2 (67%)	29 (62%)
Age range in years (minimum‐maximum)	1‐36.9	1.9‐34.4	2.8‐15.2	0.8‐30.7	15.3‐21.1	0.8‐36.9

Abbreviations: MPS, mucopolysaccharidosis; n, number of individuals.

### 3.2 Main findings

The results of posterior fossa MRI analysis are described in Table 2, according to the types of MPS. Due to the wide clinical variation, we also compare neuronopathic (MPS types I Hurler, IIA, and III) and mild neuronopathic/non‐neuronopathic (MPS types I Hurler‐Scheie, I Scheie, IIB, IV, and VI) MPS patients (Table 3).

**TABLE 2:** Posterior fossa findings in patients with mucopolysaccharidosis

Parameter	MPS type I (n = 13)	MPS type II (n = 15)	MPS type III (n = 6)	MPS type IV (n = 10)	MPS type VI (n = 3)	Total (n = 47)
*MRI‐visible perivascular spaces*						
Midbrain (n)	10 (77%)	14 (93%)	6 (100%)	7 (70%)	3 (100%)	40 (85%)
Pons (n)	3 (23%)	5 (33%)	1 (17%)	0	1 (33%)	10 (21%)
Medulla (n)	1 (8%)	3 (20%)	0	0	0	4 (8%)
Cerebellum (n)	10 (77%)	11 (73%)	0	2 (20%)	3 (100%)	26 (55%)
Mean maximum axial diameter (cm ± SD)	0.24 ± 0.06	0.26 ± 0.05	0.22 ± 0.08	0.19 ± 0.08	0.26 ± 0.05	0.24 ± 0.06
*White matter lesions*						
Midbrain (n)	0	1 (7%)	1 (17%)	0	0	2 (4%)
Pons (n)	4 (31%)	8 (53%)	1 (17%)	3 (30%)	0	16 (34%)
Medulla (n)	0	0	0	0	0	0
Cerebellum (n)	2 (15%)	6 (40%)	2 (33%)	3 (30%)	1 (33%)	14 (30%)
*Cerebellar volume*						
Normal (n)	4 (31%)	7 (47%)	3 (50%)	4 (40%)	0	18 (38%)
Atrophic (n)	2 (15%)	3 (20%)	2 (33%)	2 (20%)	1 (33%)	10 (21%)
Hypoplastic (n)	0	1 (7%)	0	0	0	1 (3%)
Apparent macrocerebellum (n)	7 (54%)	4 (26%)	1 (17%)	4 (40%)	2 (67%)	18 (38%)
*Middle cerebellar peduncle width (sagittal plane)*						
Right MCP (cm ± SD)	1.18 ± 0.25	1.15 ± 0.16	1.18 ± 0.13	1.15 ± 0.18	1.30 ± 0.10	1.17 ± 0.18
Left MCP (cm ± SD)	1.21 ± 0.22	1.18 ± 0.15	1.20 ± 0.09	1.16 ± 0.17	1.33 ± 0.06	1.20 ± 0.17
Mean (cm ± SD)	1.20 ± 0.23	1.17 ± 0.15	1.19 ± 0.11	1.15 ± 0.17	1.32 ± 0.08	1.17 ± 0.17
*Middle cerebellar peduncle width (axial plane)*						
Right MCP (cm ± SD)	1.61 ± 0.14	1.65 ± 0.17	1.55 ± 0.23	1.73 ± 0.18	1.90 ± 0.20	1.66 ± 0.19
Left MCP (cm ± SD)	1.60 ± 0.13	1.67 ± 0.17	1.53 ± 0.19	1.72 ± 0.15	1.87 ± 0.15	1.65 ± 0.17
Mean (cm ± SD)	1.61 ± 0.13	1.66 ± 0.17	1.54 ± 0.20	1.72 ± 0.16	1.88 ± 0.18	1.66 ± 0.17
*Posterior fossa shape*						
Inverted J‐shaped posterior fossa (n)	5 (38%)	10 (67%)	1 (17%)	4 (40%)	2 (67%)	22 (47%)
*Enlargement of CSF spaces*						
Absent (n)	6 (46%)	2 (13%)	2 (33%)	9 (90%)	2 (67%)	21 (45%)
Bilateral (n)	4 (31%)	13 (87%)	3 (50%)	1 (10%)	1 (33%)	22 (47%)
Unilateral (n)	3 (23%)	0	1 (17%)	0	0	4 (8%)

Abbreviations: CSF, cerebrospinal fluid; MCP, middle cerebellar peduncle; MPS, mucopolysaccharidosis; MRI, magnetic resonance imaging; n, number of individuals.

**TABLE 3:** Posterior fossa findings in neuronopathic and mild neuronopathic/non‐neuronopathic patients with mucopolysaccharidosis

Parameter	Neuronopathic MPS patients (IH, IIA, and III) n = 22	Mild neuronopathic/non‐neuronopathic MPS patients (IHS, IS, IV, and VI) n = 25	*P* value
**Male sex (n)**	16 (73%)	13 (52%)	.14^a^
**Age (y) (mean ± SD)**	7.37 ± 4.62	19.26 ± 9.84	.001^b^
**MRI‐visible perivascular spaces**			
Midbrain (n)	21 (95%)	19 (76%)	.06^a^
Pons (n)	6 (27%)	4 (16%)	.35^a^
Medulla (n)	3 (14%)	1 (4%)	.24^a^
Cerebellum (n)	11 (50%)	15 (60%)	.49^a^
**White matter lesions**			
Midbrain (n)	1 (4%)	1 (4%)	.93^a^
Pons (n)	9 (41%)	7 (28%)	.35^a^
Medulla (n)	0 (%)	0 (0%)	1^a^
Cerebellum (n)	8 (36%)	6 (24%)	.35^a^
**Enlargement of CSF spaces (n)**	16 (73%)	10 (40%)	**.02** ^a^
**Cerebellar volume**			
Normal (n)	8 (36%)	10 (40%)	.7^a^
Atrophic (n)	4 (19%)	6 (24%)	
Hypoplastic (n)	1 (4%)	0 (0%)	
Apparent macrocerebellum (n)	9 (41%)	9 (36%)	
**Sagittal plane MCP width mean (mean ± SD)**	1.15 ± 0.13	1.21 ± 0.20	.21^b^
**Axial plane MCP width mean (mean ± SD)**	1.54 ± 0.13	1.76 ± 0.14	.81^b^
**Inverted J‐shaped posterior fossa (n)**	10 (45%)	12 (40%)	.86^a^

Abbreviations: CSF, cerebrospinal fluid; IH, I Hurler syndrome; IHS, I Hurler‐Scheie syndrome; IS, I Scheie syndrome; MCP, middle cerebellar peduncle; MPS, mucopolysaccharidosis; MRI, magnetic resonance imaging; n, number of individuals.

^a^ Analysis performed by Chi‐square test for independent samples.

^b^ Analysis performed by independent *t* test.

**FIGURE 3:**
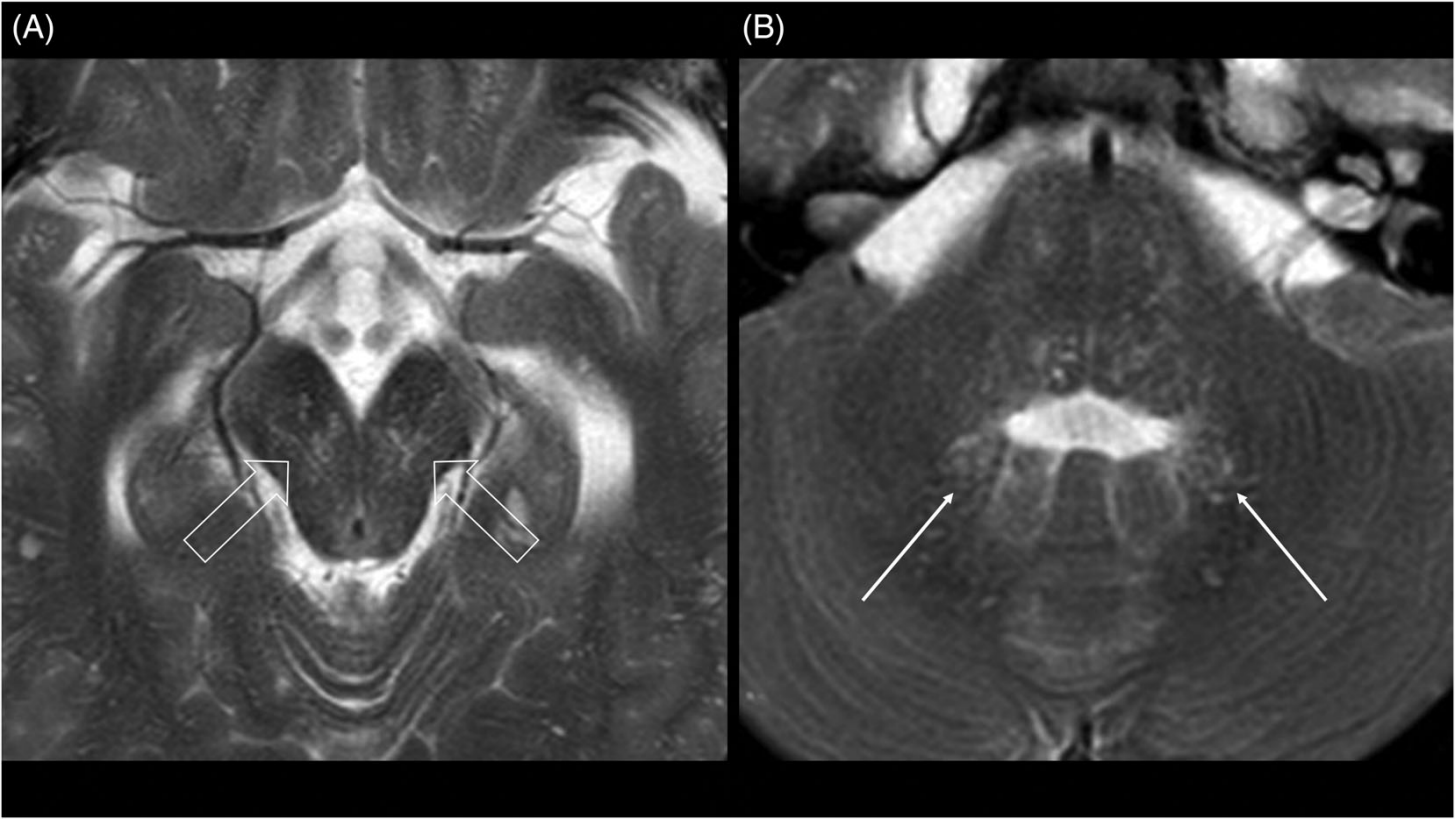
MPS IIB in a 26‐year‐old man patient. Axial T2‐weighted images show MRI‐visible perivascular spaces (A) in the cerebral peduncles (open arrows) and (B) in the white matter adjacent to the dentate nuclei (arrows). MPS, mucopolysaccharidoses; MRI, magnetic resonance imaging

MRI‐visible perivascular spaces were commonly found in the cerebral peduncles (present in 85% of individuals) (Figure 3A). The white matter adjacent to the dentate nuclei was the second most common localization (present in 55% of patients) (Figure 3B). Pons and medulla were less commonly affected (21% and 8% of individuals, respectively). The mean maximum axial diameter was 0.24 cm (SD ± 0.06 cm).

**FIGURE 4:**
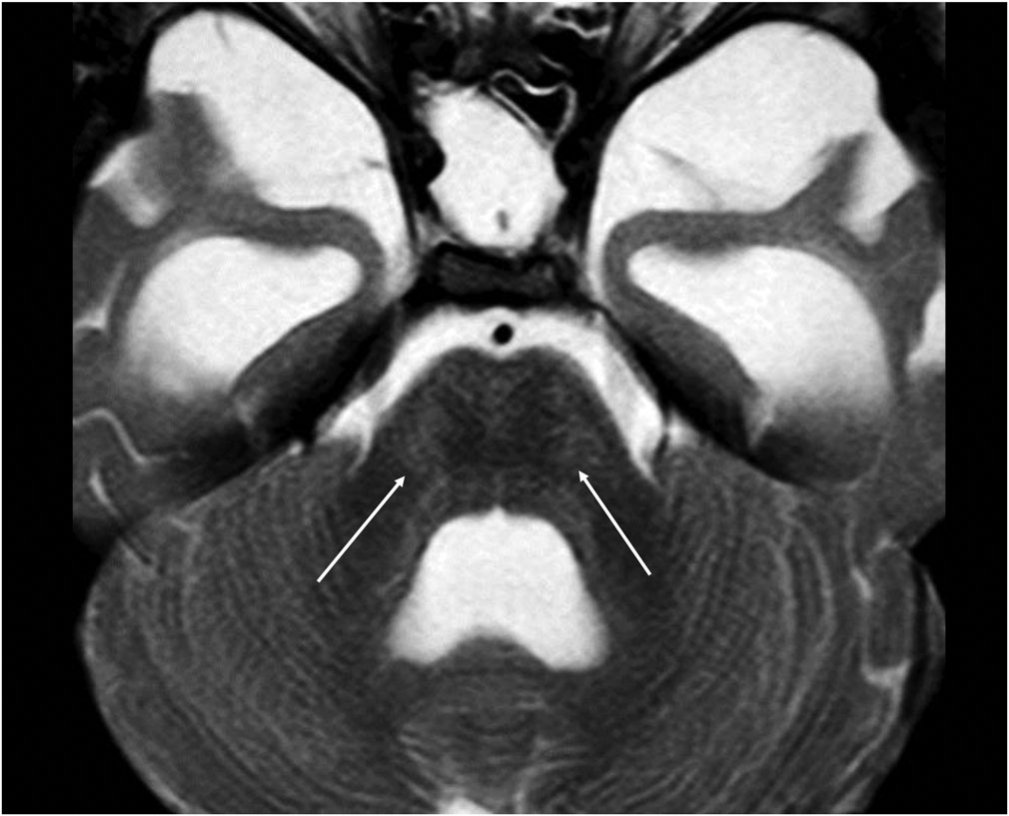
MPS IIA in a 7‐year‐old boy. Axial T2‐weighted image shows areas of slight T2‐FLAIR hyperintensity in the pons, characterizing the presence of white matter lesions. FLAIR, fluid‐attenuated inversion recovery; MPS, mucopolysaccharidoses

White matter lesions were most frequently identified in the pons and cerebellum (34% and 30% of patients, respectively) (Figure 4). Only two individuals (4%) had mild lesions in the midbrain. No case of medullary involvement was identified.

There was no statistically significant difference between neuronopathic and mild neuronopathic/non‐neuronopathic MPS patients regarding presence and distribution of MRI‐visible perivascular spaces and white matter lesions.

**FIGURE 5:**
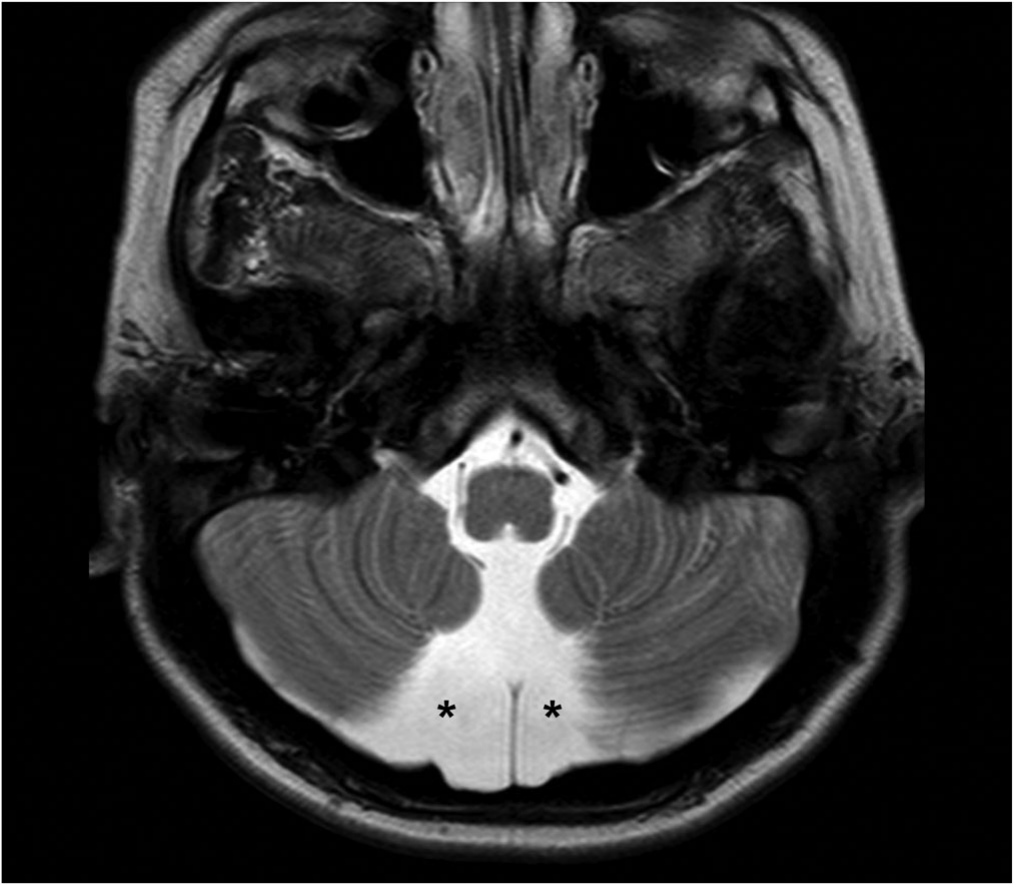
MPS IIA in a 7‐year‐old boy. Axial T2‐weighted image demonstrates enlargement of CSF spaces in posterior fossa (asterisks), inferomedially to the cerebellar hemispheres. CSF, cerebrospinal fluid; MPS, mucopolysaccharidoses

Enlargement of CSF spaces in the posterior fossa was present in 26 individuals (55%), being bilateral in 22 patients (Figure 5). All four cases of unilateral enlargement of CSF spaces were located on the left. In all cases, the enlargement of CSF spaces was situated inferomedially to the cerebellar hemispheres, next to the midline. This finding was present in 16 neuronopathic patients (73%) and only in 10 mild neuronopathic/non‐neuronopathic patients (40%), with a statistically significant difference (*P* = .02).

**FIGURE 6:**
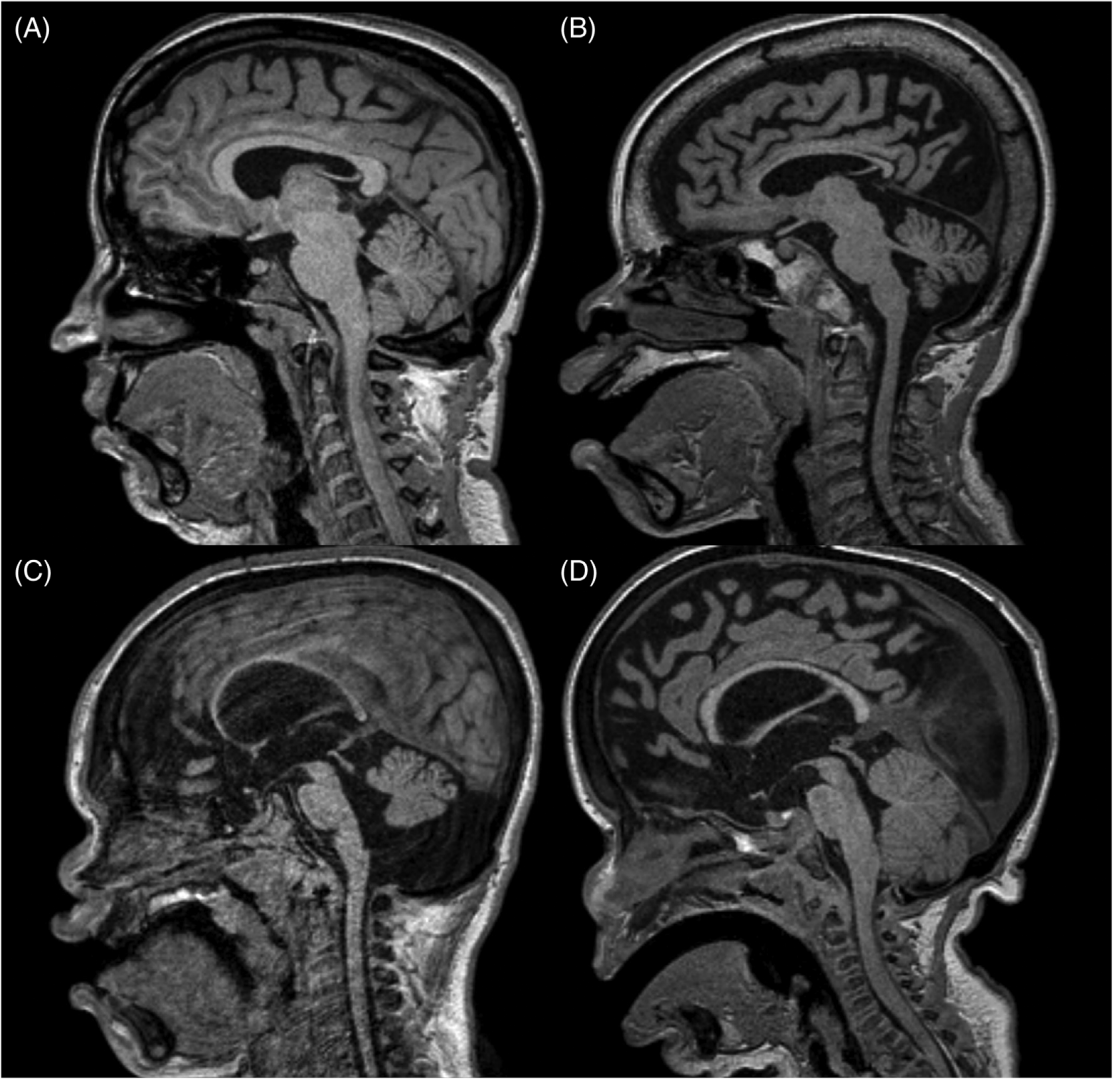
Sagittal T1‐weighted volumetric acquisition demonstrating different cerebellar volumes. A, Normal cerebellar volume in a 30‐year‐old woman with MPS I Scheie. B, Atrophic cerebellum in a 10‐year‐old boy with MPS IIIC, characterized by enlargement of cerebellar sulci. C, Hypoplastic cerebellum in a 7‐year‐old boy with MPS IIA, defined as cerebellum of reduced dimensions, but preserved morphology. D, Apparent macrocerebellum in a 4‐year‐old boy with MPS I Hurler, showing reduction of pericerebellar CSF spaces. CSF, cerebrospinal fluid; MPS, mucopolysaccharidoses

Cerebellar volume was classified as normal in 18 patients (38%). Cerebellar atrophy was present in 10 patients (21%). Apparent macrocerebellum also was identified in 17% to 67% of patients according to MPS type. Hypoplastic cerebellum was found in only one individual (3%). There was no statistically significant difference between neuronopathic and mild neuronopathic/non‐neuronopathic MPS patients regarding cerebellar volume frequencies. Illustrative examples of each cerebellum category are shown in Figure 6.

As mentioned previously, during analysis of brain images, the evaluators subjectively perceived a thickened aspect of the MCP in several individuals. Since this finding has not been previously described in patients with MPS and there is no standardized measurement of these structures, we measured MCPs in the sagittal plane based on a previous study^16^ (Figure 1A), which described a mean sagittal MCP width of 0.98 cm (SD ± 0.07 cm) in 14 control subjects with a mean age of 66.9 years (SD ± 6.5 years). In the present study, width measurement was also performed in the axial plane to provide a reference for future studies (Figure 1B). The mean MCP widths in the sagittal and axial planes in our MPS patients were 1.17 cm (SD ± 0.17 cm) and 1.66 cm (SD ± 0.17 cm), respectively. For both sagittal and axial MCP width, there was no statistically significant difference between neuronopathic and mild neuronopathic/non‐neuronopathic MPS patients.

Regarding posterior fossa shape, a depression of the posterior fossa floor in the midline sagittal plane, which we termed the “inverted J‐shaped posterior fossa,” was found in 22 patients (47%) (Figure 2), without statistically significant difference between neuronopathic and mild neuronopathic/non‐neuronopathic MPS patients.

There was no case of Chiari malformation in the study population.

### 3.3 Other analyses

Inverted J‐shaped posterior fossa was statistical significantly associated with enlargement of CSF spaces (*P* = .02) and with the presence of apparent macrocerebellum (*P* = .03). The results are shown in Table 4.

**TABLE 4:** Association between inverted J‐shaped posterior fossa and enlargement of cerebrospinal fluid spaces, and apparent macrocerebellum

Parameter	Patients with inverted J‐shaped posterior fossa n = 22	Patients without inverted J‐shaped posterior fossa n = 25	*P* value
Enlargement of CSF spaces present (n)	16 (73%)	10 (40%)	.02
Apparent macrocerebellum (n)	12 (54%)	6 (24%)	.03

Abbreviations: CSF, cerebrospinal fluid spaces; n, number of individuals.

## 4 DISCUSSION

Since most neuroimaging studies in MPS patients have focused on supratentorial findings, this study aimed to analyze and compile the main posterior fossa findings in this population. We included 47 patients with MPS types I, II, III, IV, and VI, which represents a relatively large sample, given the rarity of this condition. In summary, we found that MRI‐visible perivascular spaces and white matter lesions also occur in the posterior fossa. The former was usually small and located in the cerebral peduncles or adjacent to the dentate nuclei. The latter occurred more commonly in the pons, although more discreetly than usually seen in the supratentorial compartment.

Atrophy, a commonly described finding in the cerebral hemispheres of patients with MPS,^5^ was not the most frequent cerebellar morphology found in the present study. Approximately three‐quarters of the patients had normal cerebellar volume or apparent macrocerebellum. The evaluation of the cerebellar volume was evaluated subjectively, as usually performed in the practice of the radiologists, and as performed in a previous study with MPS patients.^12^ Considering a potential bias related to subjective evaluation, we opted for a more conservative denomination (apparent macrocerebellum), since future volumetric studies are necessary to confirm this finding.

More than half of the patients had enlargement of CSF spaces in the posterior fossa. This finding was also found in the vast majority of neuronopathic MPS patients, with a significant difference when compared to mild neuronopathic/non‐neuronopathic MPS patients. We hypothesized it may reflect a greater impact in CSF flow in more severe forms of MPS.

The lack of standardized measurement of MCP width, as well as the lack of normal reference values for this parameter, was an important limitation. In order to perform objective measurement, we used as reference parameters described in a previous study conducted in patients with Parkinson disease and multiple system atrophy, since it was the only publication we have found containing a description for MCP measurement in MR images.^16^ We are aware that additional studies with age‐matched controls are essential for correct interpretation of the values measured. Although not significant, neuronopathic MPS patients showed a slight tendency to thinner MCPs. Considering the fact that MCPs are the main cerebellar afferent pathway, it may have some relationship with severe cortical atrophy usually present in this group.^5^ It is an interesting potential finding for future investigations.

In a considerable part of the patients, we observed a pattern: a depression of the posterior fossa floor in the midline sagittal plane, which we termed the “inverted J‐shaped posterior fossa.” This finding was statistical significantly associated with enlargement of CSF spaces, and with the presence of apparent macrocerebellum, which leads us to suppose that it is related to volumetric modifications. Although they are interesting findings, we still do not know their clinical significance.

Limitations of this study include the mostly qualitative nature of our analyses. To mitigate the effect of subjectivity, all images were evaluated by consensus by two radiologists. Further quantitative studies with volumetric evaluation may add important information to the current literature.^17^ Another limiting factor is the sample size. Studies with MPS patients usually have a small sample. We included 47 patients which is a large cohort compared to those of previous studies. Nevertheless, the absolute number of participants is still small, limiting comparisons among groups and precluding analyses of subgroups.

Overall, our findings are consistent with data available in the literature, especially those of Alqahtani et al,^12^ who described the frequency of posterior fossa findings in 12 patients with MPS types I and II. Besides compiling the frequency of previously described imaging patterns, we observed potential new findings and associations for future investigations. Quantitative studies with long‐term follow‐up could help in the search for these answers.

The present study included a representative sample of MPS patients, including types I, II, III, IV, and VI, which increased the applicability of our results to MPS patients from other medical institutions.

## 5 CONCLUSION

The present study compiled the main posterior fossa findings in MPS patients. Classically described in the supratentorial compartment, MRI‐visible perivascular spaces, white matter lesions, and enlarged perivascular spaces were also found in the posterior fossa. However, atrophy, which commonly affects cerebral hemispheres, was not the most frequent cerebellar morphology found in our study. Moreover, potential findings for future research were described.

## CONFLICT OF INTEREST

The authors declare no potential conflict of interest.

## AUTHOR CONTRIBUTIONS

Conceptualization of the study: Roberta Reichert, Juliano A. Pérez, Amauri Dalla‐Corte, Filippo Pinto e Vairo, Carolina F. M. de Souza, Roberto Giugliani, Gustavo R. Isolan, Marco Antonio Stefani. Investigation and data collection: Roberta Reichert, Juliano A. Pérez, Amauri Dalla‐Corte, Filippo Pinto e Vairo, Carolina F. M. de Souza. Data analysis: Roberta Reichert, Juliano A. Pérez, Amauri Dalla‐Corte, Filippo Pinto e Vairo, Carolina F. M. de Souza, Roberto Giugliani, Gustavo R. Isolan, Marco Antonio Stefani. Supervision: Roberta Reichert, Roberto Giugliani, Gustavo R. Isolan, Marco Antonio Stefani. Original draft writing: Roberta Reichert, Marco Antonio Stefani. Manuscript review and editing: Roberta Reichert, Juliano A. Pérez, Amauri Dalla‐Corte, Filippo Pinto e Vairo, Carolina F. M. de Souza, Roberto Giugliani, Gustavo R. Isolan, Marco Antonio Stefani.

## INFORMED CONSENT

All procedures followed were in accordance with the ethical standards of the responsible committee on human experimentation (institutional and national) and with the Helsinki Declaration of 1975, as revised in 2000. Informed consent was obtained from all patients for being included in the study.
